# Evaluation of dental enamel after debonding of sapphire brackets and removal of orthodontic adhesive remnants

**DOI:** 10.1590/2177-6709.30.3.e2524266.oar

**Published:** 2025-10-20

**Authors:** Elizabety do Nascimento SILVA, Daniel Magalhães QUINTANS, Rebeca Tibau Aguiar DIAS, Frederico Barbosa de SOUZA, Rudyard dos Santos OLIVEIRA

**Affiliations:** 1 Federal University of Paraíba, Dental School (Paraíba, Brazil).; 2 Federal University of Paraíba, Dental School, Department of Chemical Engineering (João Pessoa/PB, Brazil).; 3 Federal University of Paraíba, Dental School, Department of Morphology (João Pessoa/PB, Brazil).

**Keywords:** Dental enamel, Orthodontic cracks, Microfractures, Surface roughness, Orthodontic bracket removal, Esmalte dentário, Braquetes ortodônticos, Microfraturas, Rugosidade da superfície, Remoção de braquetes ortodônticos

## Abstract

**Introduction:**

Iatrogenic effects on dental enamel are common during the debonding of aesthetic brackets and the removal of residual adhesive.

**Objective:**

To evaluate the impact of these procedures on enamel integrity via advanced imaging techniques.

**Methods:**

Human premolar crowns (n=60) were bonded with sapphire brackets and debonded using 346R pliers (G1, n=30) and 346B pliers (G2, n=30). Adhesive removal was performed with a multilaminated zirconia bur (EG1, n=30) and a multilaminated carbide bur (EG2, n=30). Surface roughness was assessed via optical profilometry; enamel loss and microcracks, via microcomputed tomography; and the adhesive remnant index (ARI), via optical coherence tomography. After normality and homogeneity tests, Mann-Whitney and Wilcoxon tests were applied (p<0.05).

**Results:**

The 346R plier presented a slightly higher ARI than the 346B plier. Both removal methods significantly increased surface roughness, without causing enamel microcracks. The zirconia bur demonstrated greater adhesive removal efficiency but was associated with increased enamel loss.

**Conclusions:**

Variations in the shape and force vector of the pliers did not affect enamel outcomes. The zirconia bur system at low speed was more efficient at removing orthodontic adhesive remnants, resulting in a lower final ARI on the enamel surface, and none of the procedures caused enamel microcracks, as evaluated by microcomputed tomography.

## RESUMO

**Introdução:**

Os efeitos iatrogênicos no esmalte dentário são comuns durante a descolagem de braquetes estéticos e a remoção de adesivo residual.

**Objetivo:**

Avaliar o impacto desses procedimentos na integridade do esmalte por meio de técnicas avançadas de imagem.

**Métodos:**

Coroas de pré-molares humanos (n=60) foram coladas com braquetes de safira e descoladas com os alicates 346R (G1, n=30) e 346B (G2, n=30). A remoção do adesivo foi realizada com uma broca de zircônia multilaminada (GE1, n=30) e uma broca de Carbide multilaminada (GE2, n=30). A rugosidade superficial foi avaliada por meio de perfilometria óptica, perda de esmalte e microfissuras por meio de microtomografia computadorizada e o índice de remanescente adesivo (IRA) por meio de tomografia de coerência óptica. Após os testes de normalidade e homogeneidade, foram aplicados os testes de Mann-Whitney e Wilcoxon (p<0,05).

**Resultados:**

O alicate 346R apresentou IRA ligeiramente maior do que o 346B. Ambos os métodos de remoção aumentaram significativamente a rugosidade da superfície sem causar microfissuras no esmalte. A broca de zircônia demonstrou maior eficiência de remoção de adesivo, mas foi associada ao aumento da perda de esmalte.

**Conclusões:**

Variações na forma e no vetor de força do alicate não afetaram os resultados do esmalte. O sistema de broca de zircônia em baixa rotação foi mais eficiente na remoção de remanescentes adesivos ortodônticos, resultando em menor IRA final na superfície do esmalte, e nenhum dos procedimentos causou microfissuras no esmalte, conforme avaliado pela microtomografia computadorizada.

## INTRODUCTION

The correction of misaligned teeth and malocclusions with fixed appliances improves oral and facial aesthetics, benefiting both the dental health and the psychological well-being of patients.^1^ Due to the increasing demand for aesthetic orthodontic treatments with less visible brackets, ceramic brackets have been developed.^2^ These brackets are classified into monocrystalline, polycrystalline, and sapphire brackets, each with specific characteristics aimed at optimizing the aesthetic and functional outcomes of orthodontic treatment.^3^

Sapphire orthodontic brackets are known for their chemical composition of pure monocrystalline aluminum oxide (Al_2_O_3_), which gives them a uniform and translucent crystalline structure. This unique composition makes sapphire brackets highly aesthetic and significantly more abrasion-resistant than stainless steel brackets.^4^ ^-^ ^6^ However, despite their wear resistance, the rigid and less tough nature of the sapphire material can lead to fracture under the pressures exerted during appliance removal, a characteristic that must be considered when choosing the best technique.^7^ ^,^ ^8^

The adhesion of sapphire brackets to dental enamel via resin adhesive systems is generally superior to that of metal brackets.^9^ ^,^ ^10^ This greater adhesion is attributed to the greater mechanical retention and, primarily, to the higher surface energy of the sapphire material, which allows for more efficient bonding with resin adhesives.^11^ This strong bond, while advantageous for the stability of orthodontic treatment, can hinder bracket removal and increase the risk of enamel damage during the debonding process.^10^ ^,^ ^12^

Recent studies have reported that due to the greater adhesion of aesthetic brackets to resin orthodontic adhesives, there may be a loss of enamel mineral substrate and microfractures and greater difficulty in removing adhesive residues with available bur and polishing systems, resulting in greater grooves in the enamel structure and even irreversible damage to the dental structure.^8^ ^,^ ^13^

Various techniques have been developed for the removal of aesthetic brackets, aiming to minimize enamel damage and facilitate the removal of adhesive residues.^14^ The technique most commonly used by clinicians is manual removal with pliers, because of its simplicity and effectiveness. However, other techniques, such as thermal removal, the use of high-power lasers, and ultrasound, are also applied in specific study situations. These alternatives offer different advantages and disadvantages, depending on the professional’s experience and the accessibility of instruments and equipment.^8^ ^,^ ^14^

Optical coherence tomography (OCT) has emerged as a valuable tool in the clinical evaluation of resin residues after debonding aesthetic brackets. This noninvasive imaging technique allows detailed visualization of enamel layers and adhesive residues without the need for X-ray radiation, assisting in determining the amount of residue present and evaluating potential damage to the enamel during the removal process.^15^ ^-^ ^18^

The present study aimed to evaluate the enamel surface after debonding sapphire orthodontic brackets and removing the remaining orthodontic adhesive. To this end, various analysis techniques have been employed, including noncontact three-dimensional optical profilometry, microcomputed tomography, and optical coherence tomography. These methodologies allowed for a comprehensive assessment of enamel integrity and the effectiveness of adhesive removal techniques, providing valuable insights for current clinical orthodontic practice.

## MATERIAL AND METHODS

This *in vitro* experimental study was approved by the Research Ethics Committee of the Health Sciences Center of the Federal University of Paraíba under number 70473123.0.0000.5188.

### SAMPLE SELECTION

The sample consisted of 60 human premolars with intact crowns extracted from patients over 18 years of age of both sexes without carious lesions or visible crown fractures. The extractions were performed following prior professional recommendations, and the donors signed donation and informed consent forms.

The sample size calculation was performed via Jamovi software (version 2.4.14; Sydney, Australia) and was based on the surface roughness variable, which was considered the primary variable of the study. The minimum difference of interest (MDI) considered was 0.5 µm, on the basis of the literature data,^8^ ^,^ ^19^ indicating that differences exceeding this value are clinically relevant for assessing changes in dental enamel. The parameters used for the calculation included an effect size of 0.8, a test power of 0.85, and a significance level of 0.05, resulting in a minimum of 28 teeth per group.

To ensure sample standardization, a surface microhardness test was performed on each sample. Three equidistant measurements were taken at the center of each sample via a microhardness tester (Knoop, Shimadzu Co., Tokyo, Japan) with a load of 490 mN for 10 seconds. The average of these three readings was calculated and used as the sample microhardness value. This value served as the inclusion and exclusion criterion for the study, with samples having values 10% above or below this average being discarded.^19^

### SAMPLE PREPARATION

The selected premolars had their crowns separated from the roots 1 mm below the cementoenamel junction via a flexible diamond disc (7012, KG Sorensen, Cotia, São Paulo, Brazil) mounted on a precision electric cutter. The crowns were embedded in acrylic resin using a PVC base, with their entire buccal surface positioned parallel to and above the resin plane. A rectangular yellow adhesive tape (4×5 mm) was applied to the middle third of the buccal surfaces of the teeth, corresponding to the bracket bonding areas. The edges of the adhesive tapes were used as reference points so that only this portion of the buccal enamel surface was exposed, ensuring better standardization of the analyses.^13^

### BONDING AND DEBONDING PROCEDURE

Before the bonding procedure of the sapphire brackets (Morelli, Sorocaba, SP, Brazil), noncontact 3D optical profilometry (Taylor Hobson, Leicester, England), optical microscopy (OM), microcomputed tomography (SkyScan 1172, Bruker, Belgium), and optical coherence tomography (Lumedica, OQ StrataScope, Durham, USA) 2D and 3D imaging were performed on all the samples.

The bonding of the sapphire brackets (Morelli, Sorocaba, SP, Brazil) followed the manufacturer’s recommendations, all with the same mesh bonding base, performed by a single orthodontist with more than ten years of experience. The enamel surfaces were conditioned with 37% phosphoric acid (Condac, FGM, Brazil) for 30 seconds, rinsed with water for 10 seconds, and then air-dried. An orthodontic adhesive orthoprimer (Morelli, Sorocaba, SP, Brazil) was applied and light-cured on the buccal surface, and an orthodontic adhesive orthobond plus color change (Morelli, Sorocaba, SP, Brazil) was used on the bracket base for bonding, following the manufacturer’s instructions. It was light-cured for 20 seconds with Radii-Cal (850 mW/cm²) (SDI Inc., Bensenville, IL, USA). The light intensity was calibrated for each polymerization using a radiometer (Demetron, Danbury, CT, USA). After the bonding procedure, the samples were stored in deionized water for 24 hours.

**Figure 1:**
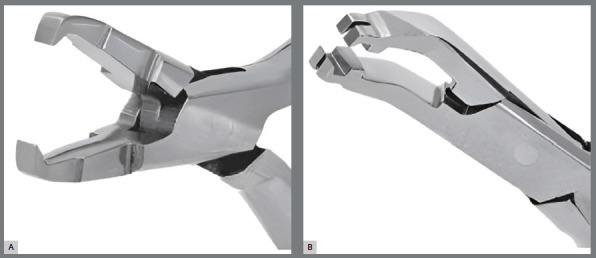
A) 346 Starlet^®^ plier; B) 346B Zatty^®^ plier.

Next, the sample was randomized via the website random.org (Trinity College, Dublin, Ireland) into two groups for debonding: G1 (n=30), which uses pliers 346R (Starlet, São Paulo, SP, Brazil) and is traditionally used for metal bracket removal and generating a supero-inferior force vector, and G2 (n=30), which uses pliers 346B (Zatty, Iacanga, SP, Brazil) and is recommended for ceramic bracket debonding, generating a lateral force vector, which may create a moment of force with a tendency toward rotational torque (Fig. 1). The debonding process was performed by a single orthodontist with more than ten years of experience. Immediately after debonding, images were taken via OCT in 2D and 3D modes, optical microscopy, and intraoral scanning to evaluate the remaining orthodontic adhesive.

### PROCEDURE FOR ORTHODONTIC ADHESIVE RESIDUE REMOVAL

**Figure 2:**
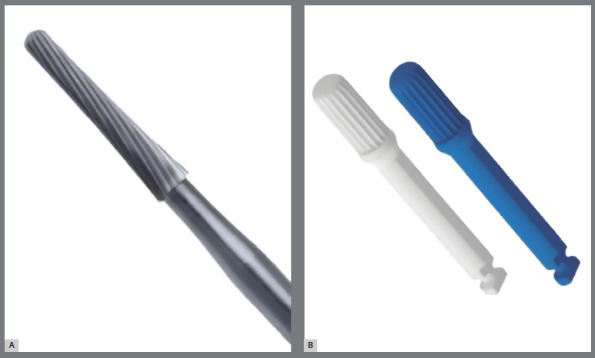
A) 36-blade carbide bur (Orthomundi^®^); B) Zirconia multilayer bur and polishing bur (Morelli^®^).

After debonding the brackets and evaluating the remaining orthodontic adhesive, the groups were randomly subdivided via the random.org website (Trinity College, Dublin, Ireland) into two new groups, each with 15 members from the initial groups: EG1 (n=30), for adhesive removal via a 36-blade carbide bur (Orthomundi, Porto Alegre, Brazil), and EG2 (n=30), for removal via a multilaminated low-speed zirconia bur (Morelli, Sorocaba, Brazil). The manufacturer of the 36-blade carbide bur did not recommend the use of a polishing system, whereas the zirconia bur manufacturer recommended the use of a fiberglass polymer polishing bur (Fig. 2). The bur was positioned parallel to the long axis of the teeth with horizontal movements, following the manufacturer’s guidelines. Sample matrices adapted to a simulation mannequin in the prosthetics laboratory of the institution proposed in this study were used to simulate ideal clinical positioning. The procedure was considered complete after visual and macroscopic assessment confirmed the satisfactory removal of the residues. A new bur was used for every ten samples, in accordance with the manufacturer’s recommendations.

After complete removal, new scans were performed via OCT in 2D and 3D modes, as well as noncontact 3D optical profilometry, microcomputed tomography, and scanning electron microscopy, to evaluate the average surface roughness and the presence of scratches, microfractures, and substrate loss in the dental enamel. These evaluations determined the efficiency of each system in removing the remaining orthodontic adhesive.

### QUALITATIVE ANALYSIS IMMEDIATELY AFTER DEBONDING

**Figure 3:**
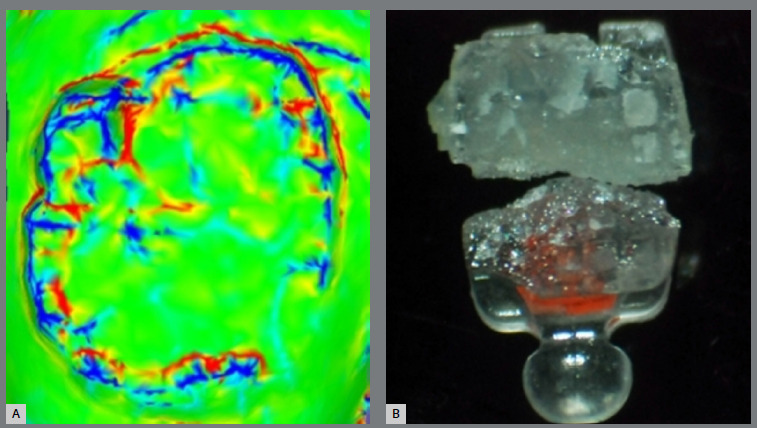
A) Image of adhesive remnant on dental enamel-MashLab^®^; B) Fractured Morelli^®^ sapphire bracket.

For the qualitative analysis of the orthodontic adhesive residue immediately after debonding, we used .STL files generated by a Cerec intraoral scanner (Dentsply Sirona, North Carolina, USA) after scanning the samples. These files were processed via MeshLab software (version 2022.02, Pisa, Italy) to evaluate the distribution and quantity of the remaining adhesive. Additionally, optical microscopy images of the sapphire brackets were taken to examine the occurrence of fractures due to the force applied by the pliers during debonding. This approach allowed for a detailed assessment of the residue adhered to the enamel and the fractures generated in the sapphire brackets (Fig. 3).

### OPTICAL COHERENCE TOMOGRAPHY (OCT) ANALYSIS

**Figure 4:**
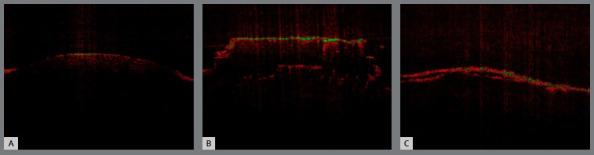
A) T0 - ARI score = 0; B) T1 - ARI score = 3; C) T2 - ARI score = 2.

The orthodontic adhesive remnant index (ARI) was evaluated via OCT at two distinct time points: immediately after debonding (T1) and immediately after adhesive residue removal with the bur system (T2), using the images obtained before bonding (T0) as a reference. ARI scores ranged from 0 to 3, where a score of 0 indicated no adhesive residue, a score of 1 indicated that less than half of the adhesive remained on the dental structure, a score of 2 indicated that more than half of the adhesive was present, and a score of 3 indicated that all adhesive remained on the tooth surface, with a distinct imprint of the bracket mesh, as shown in Figure 4.

For standardization of the tomographic evaluations, the images were analyzed by two previously calibrated examiners. An intraclass correlation coefficient (ICC) test revealed that the intraexaminer reproducibility was excellent (ICC>0.92, p<0.0001), as was the interexaminer agreement (kappa=0.9). Disagreements were resolved through consensus meetings.

### 3D NONCONTACT PROFILOMETRY ANALYSIS

The enamel surfaces were analyzed at different stages via a 3D noncontact optical profiler (Talysurf CCI Lite, Taylor Hobson). This equipment features an extended scanning range (measurement area of 6.6-75 mm), high optical resolution (0.01-0.1 Å), and lateral resolution of 0.4-0.6 μm (x- and y-axes), with scanning times ranging from 5-40 s. A repeatability of 0.02 nm (Z scan) is crucial for high-precision metrology. The measurement data were processed by TalySurf software (Taylor Hobson), ensuring the accuracy of surface characterization and quantifying the surface with more than 1 million data points in 1,024 × 1,024 pixels images.

**Figure 5:**
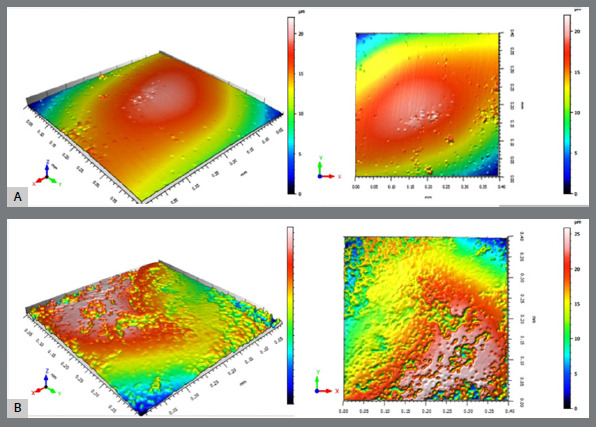
A) 3D profilometry before processing; B) 3D profilometry after processing.

To ensure analytical precision at different time points, the acrylic resin edges created during sample preparation were used as a reference. The positioning of the samples was standardized by aligning the longitudinal axis of the dental crown perpendicular to the horizontal plane of the table, and measurements were taken at the midpoint of the exposed central buccal enamel region. A comparison of the images generated at the start (T0) and end (T1) of the procedure confirmed the reproducibility of the positioning. The surfaces were analyzed to quantify the average roughness via the same protocols for T0 and T1 to determine two amplitude parameters: Sa) the arithmetic mean height of the peaks and depth of the valleys from a mean line, and Ra) the arithmetic average surface roughness (Fig. 5).

For standardization of profilometry image acquisition, the images were obtained by a single operator with more than five years of experience with the device, and an intraclass correlation coefficient (ICC) test revealed excellent intraexaminer reproducibility (ICC>0.95, p<0.0001).

### COMPUTED MICROTOMOGRAPHY ANALYSIS

The samples were scanned via an X-ray microtomograph (Skyscan 1172, Bruker, Belgium) with the following scanning parameters: 4.9 mm pixel size, 100 kV voltage, 100 mA current, 180° rotation, average of 3 measurements, 0.5° rotation step, and an Al‒Cu filter. Scans were conducted before (T0) and after the entire debonding and orthodontic adhesive residue removal process (T1).

**Figure 6:**
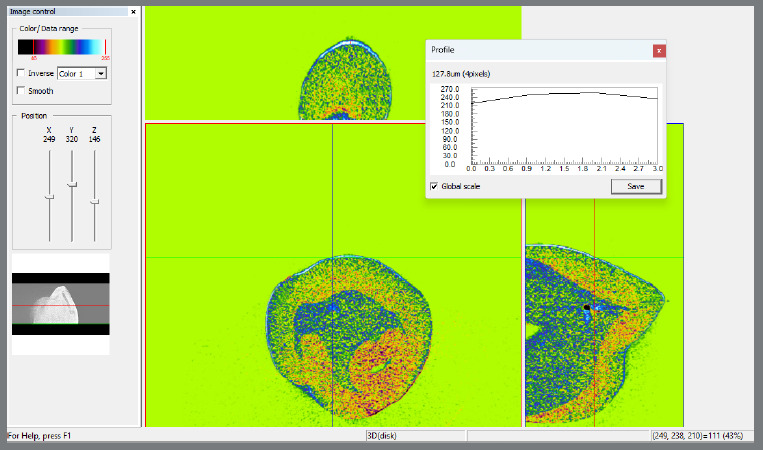
The DataViewer software window exemplifies the work with tomography.

For analysis, the volumes obtained were reconstructed in 3D via NRecon software (version 1.5.23, Bruker, Belgium) and subsequently manipulated via DataViewer software (version 1.5.1.2, Bruker, Belgium). This software allows for the overlay of images before and after bonding, debonding, and removal of orthodontic adhesive residue, generating a new dataset with coregistration. Measurements were then performed with DataViewer software at a central region of the buccal surface at three equidistant points. The average difference between the enamel from the reference images and that after the process was calculated (Fig. 6).

This approach facilitated the quantification and visualization of changes in enamel morphology and structure due to orthodontic procedures, providing detailed insights into the effects of bonding, debonding, and adhesive residue removal on dental enamel.

**Figure 7:**
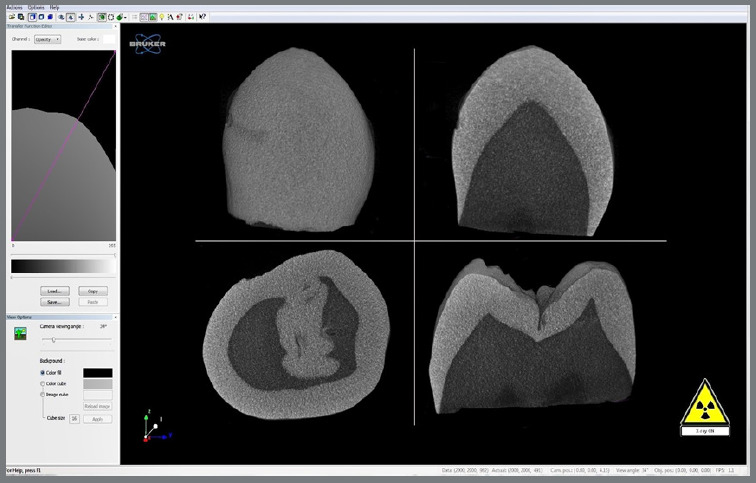
The CTvox software image shows the scan in all slices to assess the presence of microcracks.

To assess the presence of enamel microfractures due to the studied process, manipulation of the microtomographic volumes was performed via CTvox software (Bruker, Belgium) through coronal, sagittal, and parasagittal cuts, enabling a comprehensive scan of the buccal surface (Fig. 7).

For standardization of the microtomographic evaluations, images were analyzed by two previously calibrated examiners. Intraclass correlation (ICC) analysis revealed excellent intraexaminer reproducibility (ICC > 0.98, p < 0.0001) and perfect interexaminer agreement (kappa = 1.0) at two different time points, with discrepancies resolved through consensus meetings.

### QUALITATIVE ANALYSIS AFTER REMOVAL VIA SCANNING ELECTRON MICROSCOPY (SEM)

**Figure 8:**
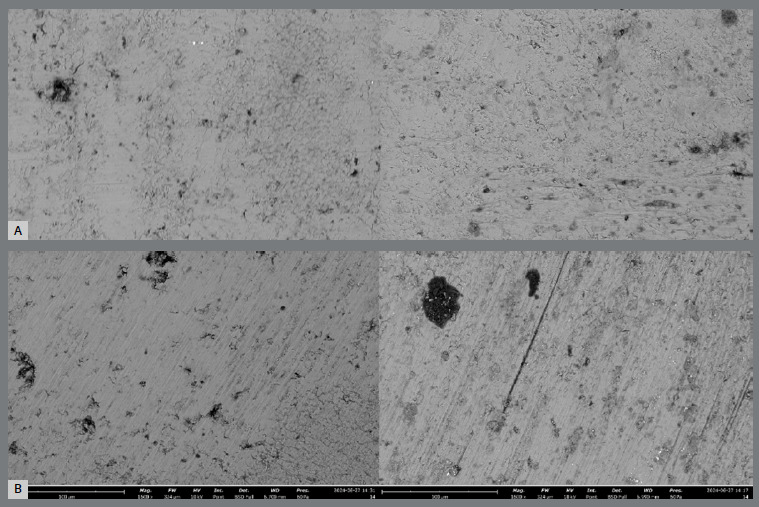
A) Representative images of EG2; B) Representative images of EG1.

Two samples from each subgroup (SG1 and SG2) were selected to represent the studied groups of orthodontic adhesive remnant removal systems. These samples were analyzed via a scanning electron microscope (Phenom, Thermo Scientific, Waltham, Massachusetts, USA) at magnifications of 160× and 1600× (Fig. 8). When there were doubts regarding the surface composition, analysis with an energy dispersive X-ray detector (EDS) was also performed.

### STATISTICAL ANALYSIS

Initially, the collected data were analyzed via descriptive statistics and the Shapiro-Wilk and Levene tests to assess normality and homogeneity, respectively. The nonparametric Mann-Whitney test was subsequently applied for unpaired comparisons between debonding pliers and their respective ARIs, between removal systems and ARIs, between removal systems and enamel substrate loss, and between removal systems and mean roughness differences. To compare the mean surface roughness of the enamel across the entire sample before and after the bonding, debonding, and removal processes, the paired Wilcoxon signed-rank test was used. All analyses, including tests of agreement and reliability, were conducted via Jamovi statistical software (version 2.4.14, Sydney, Australia), considering a two-tailed significance level of 5%.

## RESULTS

The analysis of the adhesive remnant index via OCT images at T1 revealed that, on average, the index was greater when the 346R pliers were used for debonding. However, this difference was not statistically significant (p > 0.05; Bayes factor < 1), despite the anatomical variations between the pliers and the distinct directions of force applied to the bracket by each tool (Table 1). Qualitative observations revealed that a significant majority of the brackets fractured during debonding, leaving small sapphire structure residues adhering to the orthodontic adhesive remnants on the enamel surface. Quantitatively, 66.6% of the brackets removed with the 346R pliers presented residual orthodontic device fragments, whereas 80% of those removed with the 346B pliers presented residual orthodontic device fragments.

**Table 1:** Comparative analysis Score (ARI) x Pliers / Removal System.

		95% Confidence Interval					
		Statistic	±%	p-value	Lower	Upper	Effect Size
Score (ARI) X Pliers	Bayes factor₁₀	0.493	2.49E-5				
	Mann-Whitney U	393		0.280	-1,97e−5	4.25E-6	0.127
Score (ARI) X removal systems	Bayes factor₁₀	76.1	4.24E-9				
	Mann-Whitney U	216		< 0,001	-2.00	-1.84e−5	0.520

Note. Hₐ μ 346B ≠ μ 346R / Hₐ μ Zirconia ≠ μ multi-bladed carbide.

At T2, the comparative analysis of orthodontic adhesive remnant removal via OCT images revealed that the mean scores for the group treated with the multilaminated zirconia bur were significantly lower than those for the group treated with the 36-blade carbide bur (p < 0.05; Bayes factor > 30) (Table 1).

The evaluation of enamel substrate loss on the basis of microtomographic images revealed that the multilaminated zirconia bur caused greater structural loss than did the 36-blade carbide bur (Table 2). However, the difference between the groups was not statistically significant (p > 0.05; Bayes factor between 1 and 3) (Table 3).

**Table 2:** Descriptive analysis. Removal system x Enamel loss.

								Skewness		Kurtosis	
	Group	n	SE	Median	SD	Variance	IQR	Skewness	SE	Kurtosis	SE
Enamel loss (µm)	Carbide	30	11.7	163	63.9	4082	79.3	-0.5568	0.427	21.808	0.833
	Zirconia	30	18.1	197	99.3	9860	112.3	0.0921	0.427	-0.0958	0.833

**Table 3:** Comparative analysis of removal system (zirconia/carbide) and loss of vestibular enamel structure.

					95% Confidence Interval		
		Statistic	±%	p-value	Lower	Upper	Effect Size
Enamel loss (µm)	Bayes factor₁₀	1.10	2.23E-5				
	Mann-Whitney U	351		0.143	-74.7	10.3	0.221

The 3D profilometry of surface roughness confirmed that the entire process of bonding, debonding, and adhesive removal led to a statistically significant increase in enamel roughness (p < 0.05; Bayes factor between 3 and 10) (Table 4). Comparative analysis of the two adhesive removal systems revealed that the use of a carbide multilaminated drill with 36 blades resulted in greater absolute roughness values. However, the difference between the groups was not statistically significant (p > 0.05; Bayes factor < 1) (Table 5).

**Table 4:** Comparative analysis - initial x final average roughness.

						95% Confidence Interval		
			Statistic	±%	p	Lower	Upper	Effect Size
Rm(i) μm	Rm(f) μm	Bayes factor₁₀	6.91	4.13 e-9				
		Wilcoxon W	538		0.006	-1213	-166	-0.412

Note. Hₐ μ Measure 1 - Measure 2 ≠ 0 // Rm(i) = initial average roughness, Rm(f) = final average roughness.

**Table 5:** Comparative analysis between the difference in average roughness and removal systems (carbide/zirconia).

					95% Confidence Interval		
		Statistic	±%	p-value	Lower	Upper	Effect Size
Rd(f-i) μm	Bayes factor₁₀	0.369	2.63 e-5				
	Mann-Whitney U	365		0.213	-480	1450	0.189

Note. Hₐ μ Carbide ≠ μ zirconia // Rd(f-i)= Difference between final and initial average roughness.

Qualitative analysis via SEM images corroborated these findings, highlighting more pronounced enamel striations in the group where the multilaminated carbide bur was used.

## DISCUSSION

The results of this study shed light on important aspects of enamel behavior after bonding and debonding aesthetic brackets and removing orthodontic adhesive remnants. The findings revealed that altering the shape of the pliers, and consequently the vector of force applied during the debonding of sapphire brackets, is not a determining factor in improving enamel surface outcomes. This result contrasts with similar studies in the orthodontic literature that demonstrated significant differences between conventional instruments and those specifically designed for aesthetic brackets.^8^ ^,^ ^20^ ^-^ ^24^

After the removal of sapphire brackets, the use of low-speed multilaminated zirconia burs was more efficient in removing orthodontic adhesive remnants with respect to the ARI. This finding, along with SEM images showing a greater pattern of enamel surface grooves in the multilaminated carbide bur group, contradicts the literature, which identifies high-speed multilaminated burs as the gold standard.^2^ ^,^ ^25^ ^-^ ^28^

The results of the enamel surface evaluations revealed that all methods for removing residual resin caused changes in the surface roughness of the enamel, supporting the findings of previous studies on surface roughness.^4^ ^,^ ^8^ ^,^ ^11^ ^,^ ^19^ ^,^ ^20^ However, on the basis of the current findings, a better clinical decision for removing orthodontic adhesive, which mostly adheres to remnants of fractured sapphire brackets, would be to use a low-speed multilaminated zirconia bur system, corroborating the results of a recent study.^29^

It is crucial for any removal system to be carefully monitored to balance removal efficiency with enamel preservation.^13^ ^,^ ^19^ ^,^ ^29^ In this context, a systematic review conducted in 2018^25^ indicated that both the length and width of enamel microcracks (EMCs) may increase after bracket removal, but quantitative evidence of these parameters before and after debonding is insufficient. On the other hand, strong evidence suggests that the number of EMCs likely increases after bracket debonding.^16^ ^,^ ^18^ ^,^ ^30^ This contrasts with the results obtained in this study, which did not observe the presence of microfractures in the selected human dental crowns after evaluation in the three tomographic planes of microcomputed tomography images.

The findings of this study have significant clinical implications. The choice of adhesive removal method should balance efficiency and minimize iatrogenic damage. While effective, multilaminated zirconia burs must be used cautiously to avoid excessive substrate loss. In line with this analysis, previous studies^7,30,31^ indicate that high-speed multilaminated tungsten carbide burs are effective for removing orthodontic adhesive remnants, although this removal is mostly tested after debonding metallic brackets.^10^ ^,^ ^16^ ^-^ ^17^ This does not account for the fracture process observed with aesthetic brackets.

A limitation of this study is the comparison of only two debonding methods and two adhesive removal methods. The best results in preventing enamel loss and maintaining temperature stability are obtained with high-power laser systems.^14^ ^,^ ^31^ ^,^ ^32^ However, this technique cannot be considered accessible for daily clinical use in most countries worldwide. Another alternative would be the use of ultrasound, which has shown satisfactory results in debonding, minimizing bracket adhesion and reducing the force required for removal. However, this technique requires prolonged application, causing discomfort for patients.^33^

The importance of this study for daily orthodontic practice is highlighted by the results obtained and the use of carefully selected natural human crowns, optical coherence tomography, and intraoral scanning. These methods can be clinically applied without the need for special infrastructure and have proven very effective for precise analysis of orthodontic adhesive remnants to be removed. These techniques allow for simulations closer to clinical reality and detailed evaluation of dental enamel, aiding in the selection of procedures that minimize damage and preserve oral health.^15^ ^-^ ^18^ ^,^ ^26^ ^,^ ^32^

Thus, this study contributes to the body of knowledge on the effects of bonding and debonding procedures of advanced aesthetic brackets on dental enamel, emphasizing the need for careful techniques to minimize damage and ensure effective adhesive removal. Preserving enamel health must remain a priority in orthodontic practice, ensuring that the benefits of less visible orthodontic treatments are not compromised by iatrogenic damage.

## CONCLUSIONS

The alteration of the plier shape of ceramic brackets to change the debonding force vector is not decisive for achieving better results on the enamel surface.

The removal system using low-speed multilaminated zirconia burs is more efficient in removing orthodontic adhesive remnants after debonding sapphire brackets in terms of the final ARI on the enamel surface.

The bonding, debonding, and adhesive removal processes increased enamel surface roughness, but no microfractures were detected in the enamel structure of the studied human dental crowns.
